# Purulent Bronchitis Complicating Measles and Rubella

**Published:** 1919

**Authors:** 


### Purulent Bronchitis Complicating Measles and Rubella.

By Temp. Lieut.-Col. W. M. Macdonald, N. Z. M. C., Major T. R. Ritchie, N. Z. M. C., and Lieut. J. C. Fox, R. A. M. C. *The British Medical Journal,* November 2, 1918.

The authors give in detail a series of cases of exanthematous diseases complicated by purulent infection of the bronchi, and conclude that purulent bronchitis is a condition in which there is a definite symptom-complex resulting from a multiple pulmonary infection, but that the actual germs affecting this symbiosis are subject to variations. This places purulent bronchitis in the category of such conditions as hemolytic jaundice, pernicious anemia, Banti's disease, typhoid and malignant neoplasm, in all of which a definite clinico-pathological condition is recognized as being of varied and multiple origin.

*Clinical Course.* In most cases the onset was that of an ordinary cold, in others there had been a bronchial and nasal catarrh for a week or longer, while in a few cases the men complained of having had a sore throat and severe cough for a month or so beforehand. Headache was a common feature and in a few cases there was profuse sweating.

*Temperature.* The temperature was usually high at the outset, and in the lighter cases it dropped by lysis in a few days. In severe cases it followed no definite course. There was frequently an antemortem fall of temperature in the 24 or 36 hours before death.

*Rash.* The eruption was mobiliform in some cases; in others it clearly resembled in appearance and distribution that of rubella; while in a few it was scarlatiniform. In many of the cases Koplik's spots were present and the rash sometimes became petechial. A marked feature was the development of a secondary or a tertiary rash, of septic origin, which could not be ascribed to any factor employed in the treatment. In 2 of the cases observed, the patients developed measles, and in 2 others scarlet fever, during convalescence from rubella.

*Expectoration.* With very few exceptions the measles and rubella cases had purulent expectoration, but about 10 o/o of these cases never became really ill. In the unfavorable cases expectoration ceased some 24 or 36 hours before death, and the bronchi became filled with pus. In a few cases the sputum was rusty, in a few streaked with blood, while one or two had definite hemoptysis or epistaxis.

*Cough, Respirations, etc.* The cough was frequently hacking and persistent. Usually there was a marked tachypnea from the outset without much distress.

*Pulse.* The pulse was usually rapid from the outset, the volume and tension were low but the pulse was not definitely dicrotic. Even where the lung involvement was not extensive, the heart seemed to be affected in an extreme degree by toxemia.

*Lividity.* Extreme lividity of the face, ears, head and trunk came on in severe cases before the heart was affected. Only very few cases which presented this feature recovered.

*Throat, etc.* In the severe cases there was usually a very dirty condition of the mouth and throat, and the tongue became dry, brown, and covered with sores. A well marked laryngitis causing disphonia or complete aphonia was present in about 80 o/o of the cases.

*Nervous System.* This was usually affected early and deeply.

*Toxemia.* Albuminuria, when present, was slight. A very definite mousy odor, such as is found in typhus, was present in most of the fatal cases.

*Complications.* Conjunctivitis occurred in some cases and in a few was purulent. Two cases developed otitis media and two had trombosis affecting one leg.

*Physical Signs.* The most striking feature of the physical examination in the early stages was the absence of signs suggesting a marked or extensive involvement of the lung tissues in patients who presented all the clinical features of a severe pneumonia.

*Treatment.* 1. *Drugs.* Heart stimulants, such as brandy, strychnine and digitalis, ammonium carbonate, oxygen warmed and alcoholized, stimulant expectorants and antiseptic inhalations have been tried. None of these had any effect on the general course of the disease.

2. *Vaccines.* 3 vaccines were used, and it must be noted that no one of these included all the organisms which appear to be responsible for the clinical symptoms. These vaccines were : *a*) A mixed stock vaccine consisting of 1,250 million Pfeiffer's bacillus and 250 million Friedlander's bacillus per cc. *b*) A vaccine consisting of 2 preparations containing : streptococcus lanceolatus and streptococcus pyogenes longus. *c*) A vaccine consisting of 2 preparations containing : staphylococcus aureus, pneumococcus, streptococcus and a Gram-negative bacillus.

*General Treatment.* The cases were nursed in roomy, well ventilated wards, and although the weather was cold, it was considered that fresh air was of more importance than warmth, and the windows were always kept well open.

*Bacteriology and Pathology.* 1) *Sputum.* The organisms constantly present in sputum were : streptococci, staphylococcus aureus and B. influenzae. Among other organisms less frequently met with were micrococcus catarrhalis, B. fusiformis, diphtheria bacilli, pneumococcus, diplococcus crassus and staphylococcus albus.

2) *Blood Cultures.* Careful investigation of organisms isolated from the blood of several patients during life showed that streptococci were almost exclusively and constantly present.

3) *Blood Counts.* In place of the marked leucocytosis to be expected in the presence of a bacteremia, the typical count seemed to lie between 8,000 and 12,000 per cc.

4. *Bacteriological Examination of Material obtained Postmortem.* The majority of the cultures were typically streptococcal, though staphylococcus aureus was present in several cases. Only in a few cases was B. influenzae isolated in pure culture.

*Inoculation Experiments.* Streptococci from the spleen, pericardial fluid and blood have been shown to be lethal to mice in moderate doses. In the case of both the streptococcal types already mentioned, death occurred 4 to 6 days after injection. The organisms were recovered from the blood and spleen after death, and were found in abundance at the point of injection where an accumulation of pus occurred.

*Postmortem Conditions.* — *Pleura.* In the majority of cases, a greater or lesser degree of fibrinous or sero-fibrinous pleurisy was present. As a rule, the accumulation of fluid was very small. The condition was usually more marked on the right side.

*Lungs and Bronchi.* In the advanced cases the following conditions were usually found in basifugal succession :

1. A basal consolidation.

2. A zone of slightly aerated lung intermediate between lobar pneumonia and bronchopneumonia.

3. A belt of bronchopneumonia.

4. A belt with minute consolidation elements around the bronchi closely dotted over the congested and oedematous lung.

5. An upper region of simple bronchitis.

*Heart.* In a number of cases there was marked sero-fibrinous pericarditis.

*Spleen.* In the majority of cases the spleen was normal, showing but a tendency to become flattened.

The authors record a series of cases of purulent bronchitis observed at Aldershot, which they compare with the infection studied at Camp Sling. The outstanding feature of this series of reports is the almost universal association of measles and rubella with a streptococcal bronchitis or septicemia.

